# Sub-aortic tunnel stenosis complicated by infective endocarditis: a case report

**DOI:** 10.11604/pamj.2022.41.165.29866

**Published:** 2022-02-24

**Authors:** Messaoudi Yosra, Nejeh Ben Halima

**Affiliations:** 1 Université de Sousse, Faculté de Médecine de Sousse, 4002, Sousse, Tunisie,; 2 Department of Cardiology Ibn El Jazzar Hospital, 3100, Kairouan, Tunisia,; 3 Laboratory of Research «Heart Failure LR12SP09», 4000, Sousse, Tunisia

**Keywords:** Congenital heart disease, sub-aortic tunnel, endocarditis, echocardiography, case report

## Abstract

Tunnel sub-aortic stenosis is a very unusual form of obstruction to left ventricular outflow. Endocarditis involving this congenital anomaly is extremely rare and not described in literature. We report the case of a 45-year-old woman who was diagnosed with a severe left ventricle outflow obstruction because of sub-aortic tunnel and was proposed for elective surgery. One month after diagnosis of her heart disease, she presented with fever and deterioration of general condition. Diagnosis of infective endocarditis secondary to a community acquired streptococcus viridans was confirmed on the basis of biological and echocardiographic abnormalities. The evolution was fatal because of multivalvular destruction and severe left ventricle dysfunction, despite rapid surgery and antibiotherapy. In this case report, we describe an extremely rare situation and we insist on the aggressive character of infective endocarditis in sub-aortic tunnel. We also emphasize the importance of antibioprophylaxis in this specific condition.

## Introduction

Sub-valvular aortic stenosis is a common adult congenital heart disease. It represents the second most common type of aortic stenosis with a prevalence of 6.5% [1]. It encompasses a variety of anatomic lesions: sub-aortic membrane which is the most frequent type (75% to 85%), thick fibromuscular ridge or fibromuscular channel along the Left Ventricle Outflow Tract (LVOT) (tunnel or tubular) [2]. Tunnel LVOT obstruction is extremely rare. Also, to the best of our knowledge, no case of infective endocarditis involving sub-aortic tunnel has been described. We present a case of severe endocarditis with multi-valvular destruction in a 45-year-old woman known to have a severe sub-aortic stenosis.

## Patient and observation

**Figure 1:**
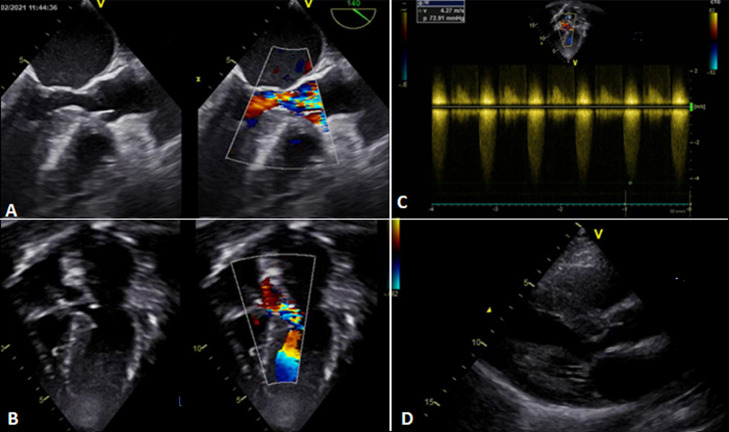
severe sub-aortic stenosis on echocardiogram with significant turbulence in the left ventricle outflow tract (A, B); and a peak instantaneous pressure gradient of 80 mmHg (C). Significant hypertrophy of the left ventricle as a consequence of the severe sub aortic stenosis (D)

**Patient information:** a 45-year-old woman presented to our department of cardiology because of fatigue, loss of appetite and excessive sweating lasting for 15 days. She had a recent diagnosis of severe sub-aortic stenosis because of sub-aortic tunnel. Her heart disease was diagnosed accidentally one month ealier because of murmur heart discovered on routine examination by her family doctor. Echocardiographic examination revealed significant turbulence in the LVOT because of a sub-aortic tunnel and the peak instantaneous pressure gradient was estimated to 80 mmHg (Figure 1). Aortic valve was normal. The existence of a sub-aortic tunnel was confirmed during cardiac catheterization, where a peak to peak pressure gradient of 90 mmHg was demonstrated. Elective surgery was scheduled.

**Clinical findings:** on examination, she was well oriented lying on the bed comfortably. Her blood pressure was 130/70 mmHg, pulse was 100 beats/min; her respiratory rate was 20/min. She was febrile (38.5°C). Cardiac auscultation revealed the systolic ejection murmur at the right upper sternal border which radiated to the neck with a diastolic murmur. There were no other abnormalities mainly neither localizing neurologic signs nor cutaneous lesions.

**Timeline of current episode:** on meticulous examination of her medical history, it was learned that she had a tooth extraction 3 weeks ago and no antibiotic prophylaxis was given. Thus, a dental portal of entry was highly suspected.

**Figure 2:**
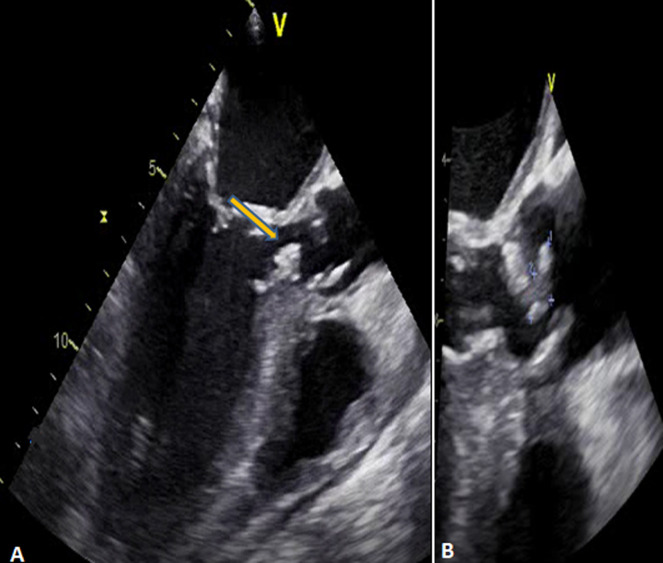
vegetations on the sub-aortic tunnel and aortic valve; transoesophageal echocardiogram 3 chamber view 120°: a thickening and an oscillating vegetation is observed in the LVOT (yellow arrow) (A); a 10*6 mm vegetation is evidenced across the aortic valve (B)

**Diagnostic assessment:** laboratory investigations revealed hemoglobin 12 g/dl, leucocytosis (26x10^6^/l), platelets 500x10^3^/l and raised C reactive protein (250 mg/l), serum sodium 135 mmol/l, serum potassium 4.3 mmol/l, blood urea 4.5 mmol/l and serum creatinine 98 μmol/l. cytobacteriological analysis of urine was normal. Chest X-ray revealed cardiomegaly. Twelve lead ECG showed sinus tachycardia with features of left ventricular hypertrophy. The diagnosis of infective endocarditis was suspected and transoesophageal echocardiogram showed irregular thickening of the anterior and posterior aspect of the sub-aortic tunnel with frond-like vegetations, protruding along the LVOT (Figure 2). The aortic valve was severely damaged with one vegetation of 10*6 mm and mild regurgitation.

**Diagnosis:** thus diagnosis of infective endocarditis involving the sub-aortic tunnel stenosis and the aortic valve was retained.

**Figure 3:**
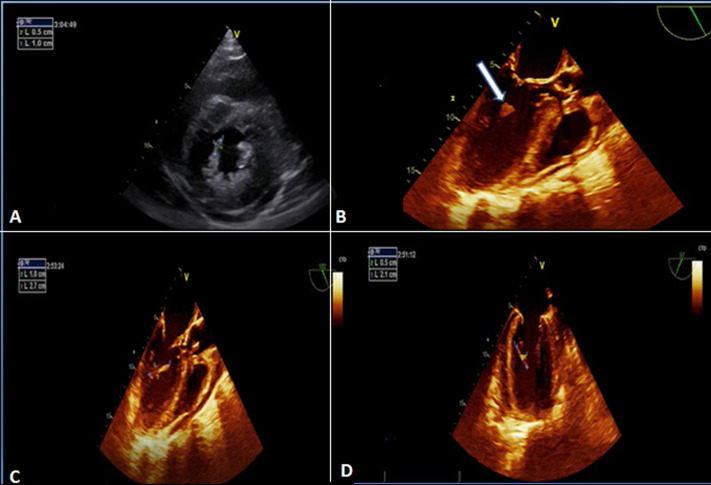
vegetation on the mitral valve on echocardiogram; transthoracic echocardiogram: vegetation on the posteromedial mitral valve pillar shown in short axis view (A); this vegetation was more studied on transoesophageal echocardiogram (B, C, D)

**Therapeutic interventions:** because blood cultures were negative, she was treated with empirical antibiotherapy based on intravenous gentamicin, and vancomycin as she was allergic to penicillin. Transoesophageal echocardiography done 48 hours later because of persistent fever showed extension of the infection to the mitral apparatus with another vegetation on the anterior mitral valve leaflet and on the posteromedial papillary muscle (Figure 3). Multidisciplinary decision was urgent surgery. She had resection of the sub-aortic obstruction, dual mitro-aortic valve replacement and she was left under extracorporeal membrane oxygenation (ECMO) because of severe left ventricle dysfunction.

**Follow-up and outcome of interventions:** however, the cardiovascular status of the patient did not improve as she remained in cardiac shock, and 72 hours after surgery she had a cardio respiratory arrest and died. Culture of the vegetation grewed sensible streptococcus viridians.

**Patient perspective:** the parents of the patient (her mother and brother) were convinced with the gravity of the situation and satisfied with the treatment despite the unfavorable outcome.

**Informed consent:** a written informed consent was obtained from the patient for the publication of details, which can include photographs and/or videos and/or case history to be published in any printed/online journals.

## Discussion

Infective endocarditis complicating left ventricle outflow tract stenosis is a poorly known entity. Early reports emphasized the association of sub-aortic stenosis and infective endocarditis particularly in patients suffering from hemodynamically severe obstruction [3-5]. However, recent literature is extremely poor. To the best of our knowledge, this is the first case of a patient with vegetation involving a sub-aortic tunnel reported in the English and French literature. In this case report, two main mechanisms of infectious lesions are possibly involved: The infectious process may originate on the aortic valve, or on the obstructing tunnel. There were vegetations in both sites in our case at initial presentation. However, the first hypothesis is that aortic valve is the starting point of infection. In fact, the high velocity jet of blood passing through the stenotic sub-valvar orifice impinges upon it during systole and is responsible for thickening and distortion of the aortic valve causing the endothelial trauma which is the starting lesion of infective endocarditis. Although bacterial endocarditis may have developed on a previously normal aortic valve, it is more likely that the infection developed on previously damaged endothelium.

Regarding the second hypothesis, echocardiographic findings in our case suggest that the infectious process originated in the center of the tunnel with a centrifugal effect of the turbulent flow causing dissemination of the infection in the aortic valve, the anterior mitral leaflet valve and even in the sub-valvular mitral apparatus. It is not excluded also that like hypertrophic cardiomyopathy, pathogenis of infective endocarditis can be explained by endocardium damage of the mitral or aortic valve, consequence of turbulence of blood flow during ejection and of the contact between the mitral anterior leaflet and the septum during systole as well as mitral regurgitation [6].

Whatever the mechanism is, there is no doubt that when infective endocarditis occurs the prognosis is considered to be poor. Infection was fatal, rapidly progressive inspite of a common and susceptible streptococcus. It was characterized by severe mitral and aortic damage, heart failure and deterioration in the hemodynamic status, which indicated an urgent surgery.

Current European and American guidelines don´t recommend antibiotic prophylaxis as it was considered that native valvular or sub-valvular stenosis is not one of the underlying cardiac conditions with a high risk for infective endocarditis even though data regarding this issue are scarce [7,8]. Considering our case together with the fact that recent published data have shown a significant rise in the incidence of total IE after antibioprophylaxis was restricted, it seems appropriate to reconsider the antibioprophylaxis administration in sub-aortic stenosis patients, at least before dental procedures mainly in our African context where evidence from prospective, randomized, controlled trials is still lacking.

## Conclusion

Infective endocarditis is a rare occurrence in sub-aortic tunnel stenosis. Nevertheless, it seems that it occurs particularly in patients suffering from severe obstruction and is responsible for multiple valve involvement, local extension and very poor prognosis. We would like to stress the need for vigilance and adequate prophylaxis against infective endocarditis in patients with sub-aortic obstruction.
